# Homogeneous reservoir computing system based on CMOS-compatible optoelectronic synapses and memristors

**DOI:** 10.1080/14686996.2026.2718043

**Published:** 2026-08-27

**Authors:** Shaopeng Feng, Kai Li, Senhao Yan, Yunhao Luo, Xiaomin Cheng, Xiangshui Miao

**Affiliations:** School of Integrated Circuits, Hubei Key Laboratory for Advanced Memories, Wuhan National Laboratory for Optoelectronics, Huazhong University of Science and Technology, Wuhan, China

**Keywords:** Optoelectronic synapses, memristors, reservoir computing, homogeneous, neuromorphic vision system

## Abstract

Dynamic image detection and recognition are critical in machine vision. In traditional visual systems, sensing, storage, and computing are usually separated, leading to significant data transfer overhead. In this work, we presented a homogeneous silicon-based reservoir computing (Si-RC) system, which is composed of SiN_x_ optoelectronic synapses and memristors for the real-time recognition of dynamic images. ITO/SiN_x_/Pt optoelectronic synapses demonstrated excitatory postsynaptic current (EPSC) and paired-pulse facilitation (PPF) behaviors, functioning as virtual nodes in the reservoir to encode the temporal features of optical image signals. Ti/SiO_2_/SiN_x_/Pt memristors showcased uniform resistive switching and electronic synaptic properties, with a coefficient of variation as low as 0.90% in long-term potentiation/depression after applying 10^4^ consecutive voltage pulses. The Ti/SiO_2_/SiN_x_/Pt memristors were utilized as trainable weights in the readout layer of Si-RC system, enabling learning and inference. The Si-RC system sensed and preprocessed ultraviolet signals from rocket exhaust plumes then identified the rocket’s movement directions, achieving 100% recognition accuracy. Compared to multilayer perceptron network, the system demonstrated a 1.9-fold improvement in training speed.

## IMPACT STATEMENT

In this study, we report a fully silicon-based, homogeneous reservoir computing (Si-RC) system integrating ITO/SiN_x_/Pt optoelectronic synapses and Ti/SiO2/SiN_x_/Pt memristors for real-time sensing and recognition of dynamic ultraviolet (UV) images. To address the significant integration complexity, high fabrication cost, and severe CMOS incompatibility of traditional heterogeneous neuromorphic systems (often relying on 2D materials, organic compounds, or carbon nanotubes), we leverage mature, CMOS-compatible silicon nitride/oxide dielectric platforms to construct both the reservoir preprocessing nodes and the trainable readout memory weights within a unified material ecosystem.

## KEY HIGHLIGHTS AND NOVELTIES

- **Fully Homogeneous Silicon-Based Platform**: First demonstration of a monolithic, CMOS-compatible Si-RC system where both optoelectronic synaptic nodes and non-volatile memristors are fabricated using standard silicon oxide/nitride processing, offering exceptional potential for large-scale hardware deployment.
- **Trap-Mediated Dynamic Photonic Memory**: The ITO/SiNx/Pt optoelectronic synapses exploit Si dangling bonds to achieve pronounced EPSC and PPF behaviors under 365 nm UV light, successfully transforming temporal optical inputs into distinguishable feature photocurrents without requiring complex image cropping or binarization.
- **Ultra-Uniform Weight Tuning**: The Ti/SiO2/SiNx/Pt memristors feature forming-free resistive switching, 16 distinct retention states at 85°C (10^3^ s), and superior LTP/LTD endurance over 10^4^ voltage pulses with an unprecedentedly low variation coefficient (Cv) of 0.90%.
- **High Performance & Rapid Convergence**: The integrated Si-RC system achieves 100% recognition accuracy for rocket plume movement directions with a 1.9-fold speedup in training iterations compared to traditional software MLP networks.

## GRAPHICAL ABSTRACT

**Figure:**
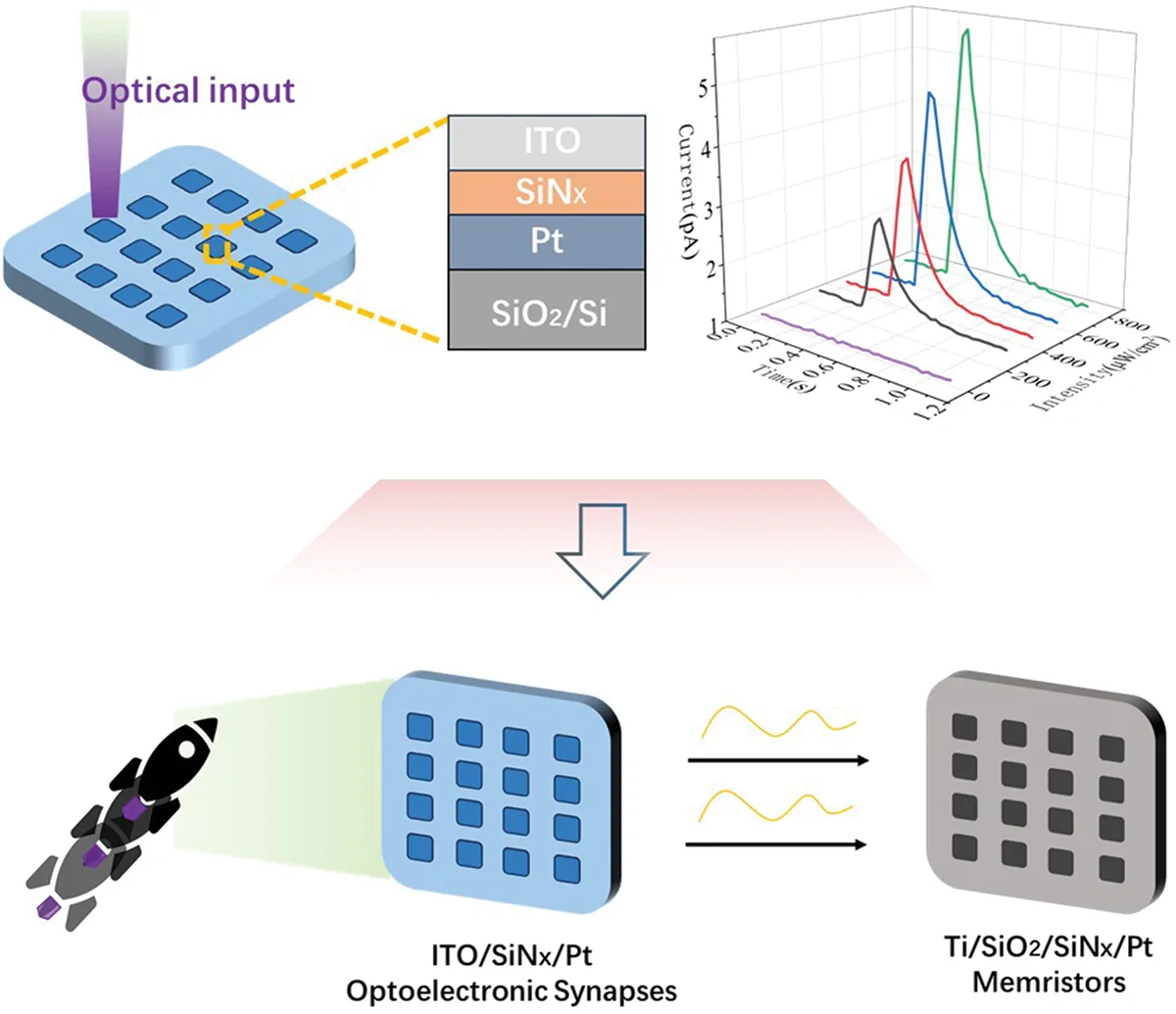

## Introduction

**Figure 1.**
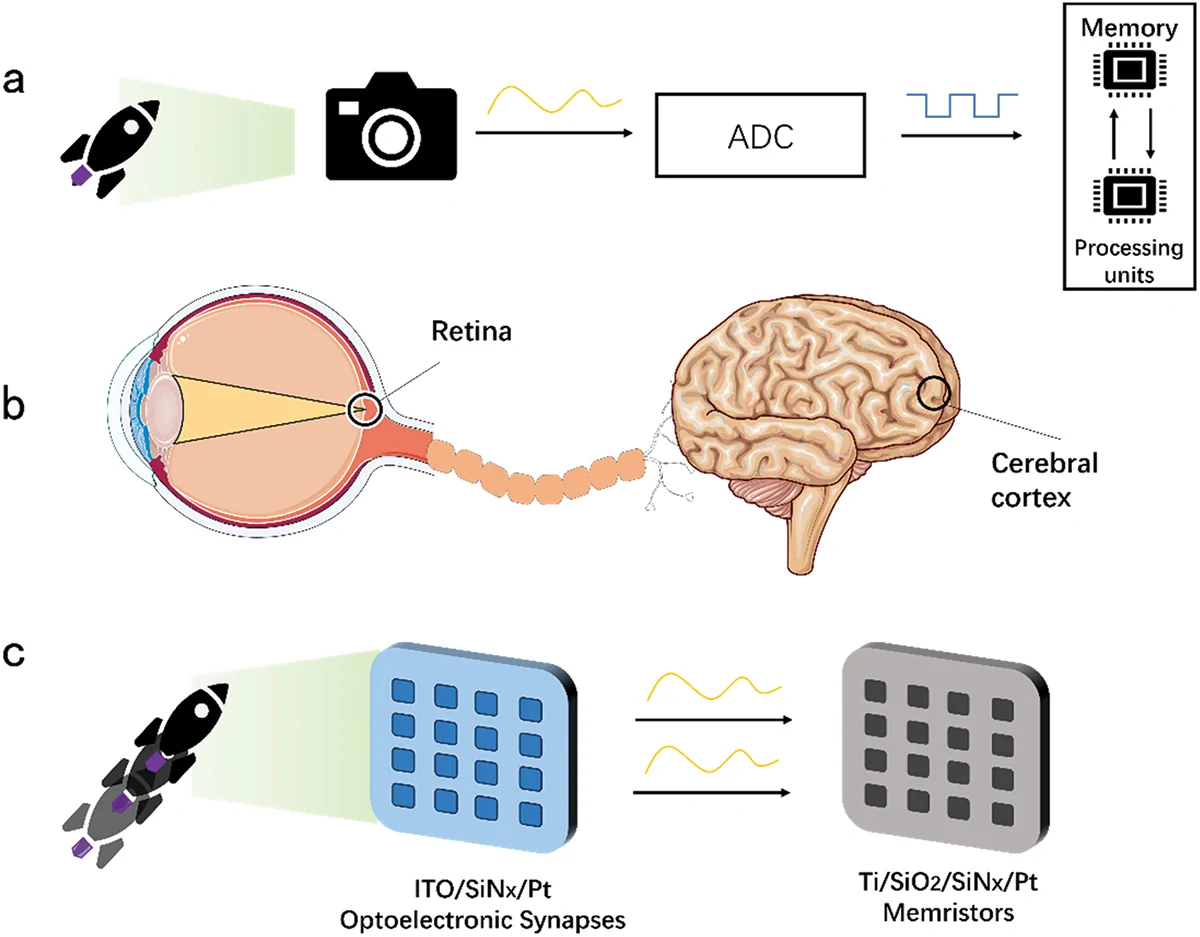
Reservoir computing system based on SiN_x_ optoelectronic synapses and memristors: (a) Optical image processing scheme in conventional machine vision. (b) Schematic of the human visual system. (c) Schematic diagram of the proposed Si-RC system for recognizing dynamic images with temporal information.

Ultraviolet (UV) sensors have shown significant application potential in fields such as astronomical observation, rocket guidance, industrial inspection, and UV imaging [1–3]. As illustrated in Figure 1(a), conventional UV machine visual systems usually operate with physically separated sensing, memory, and processing units. This architecture not only increases signal processing latency and system complexity but also leads to high energy consumption due to frequent data transfer [4,5]. As shown in Figure 1(b), the human visual system receives and preliminarily processes images through the retina before transmitting the information to the cerebral cortex for advanced processing and memory storage [6]. Inspired by human vision, neuromorphic vision systems based on reservoir computing (RC) networks have been widely explored for their ability to simultaneously sense and process images while requiring training only at the readout layer, offering an energy-efficient solution for visual information processing [7–9]. In RC system, optoelectronic synapses serve as nodes in the reservoir layer, where they sense and process optical signals with temporal information, and the extracted temporal features are then passed to the readout layer for recognition [10,11]. Memristors, leveraging in-memory computing strategies, can serve as the weights of the readout layer for training and inference, substantially reducing data movement and energy overhead [12–15]. Compared with conventional machine visual systems, neuromorphic vision systems based on optoelectronic synapses and memristors offer reduced training and data transfer requirements, and thus hold great promise for building efficient visual systems.

The illustrations in (b) were adapted from Servier Medical Art, licensed under CC BY 4.0.

However, existing optoelectronic synapses are usually based on two-dimensional materials [16–19], organic compounds [20–23], graphene [24–27], or carbon nanotubes [28–31], which face challenges in terms of CMOS compatibility and large-scale manufacturability. Moreover, optoelectronic synapses and memristors in RC system are usually fabricated by heterogeneous materials and fabrication processes, further increasing system complexity and fabrication cost [4,5,5,5,32]. Silicon oxide/nitride, with their mature fabrication processing and cost-effectiveness, remain key materials in the semiconductor industry. Developing optoelectronic synapses and memristors based on silicon oxide/nitride represents a promising and innovative direction. Such a homogeneous silicon-based RC system offers potential for mass fabrication and practical deployment in neuromorphic vision applications. Compared with previously reported heterogeneous reservoir computing systems that integrate optoelectronic synapses and memristors based on different material platforms, the proposed homogeneous silicon platform offers several distinct advantages, including simplified fabrication processes, improved CMOS compatibility, reduced integration complexity, and enhanced potential for large-scale manufacturing. Therefore, developing a homogeneous silicon-based reservoir computing system is an important step toward practical neuromorphic vision hardware.

In this work, we developed a Si-RC system consisting of ITO/SiN_x_/Pt optoelectronic synapses and Ti/SiO_2_/SiN_x_/Pt memristors for recognizing the movement directions of a rocket, as shown in Figure 1. The ITO/SiN_x_/Pt array encodes the temporal features of UV signals from the rocket’s exhaust plume, utilizing dynamic images that do not require cropping or binarization. The Ti/SiO_2_/SiN_x_/Pt memristors exhibit long-term synaptic behavior and serve as the weights of readout layer for training and inference. The Si-RC system achieved 100% recognition accuracy and a training speed 1.9-fold that of software-based multilayer perceptron (MLP).

## Results and discussion

### Short-term synaptic behaviors of the SiN_x_ optoelectronic synapses

In RC visual system, the reservoir layer is composed of randomly connected optoelectronic synaptic devices, which are responsible for encoding the temporal features of input signals. To fulfill this role, optoelectronic synapses must exhibit nonlinear decaying memory behavior, enabling the integration of visual sensing and signal processing functionalities [11,33–35]. Owing to the intrinsic defect states and the wide bandgap (~5.0 eV) [36,37], SiN_x_ is a promising material for UV optoelectronic synapses.

**Figure 2.**
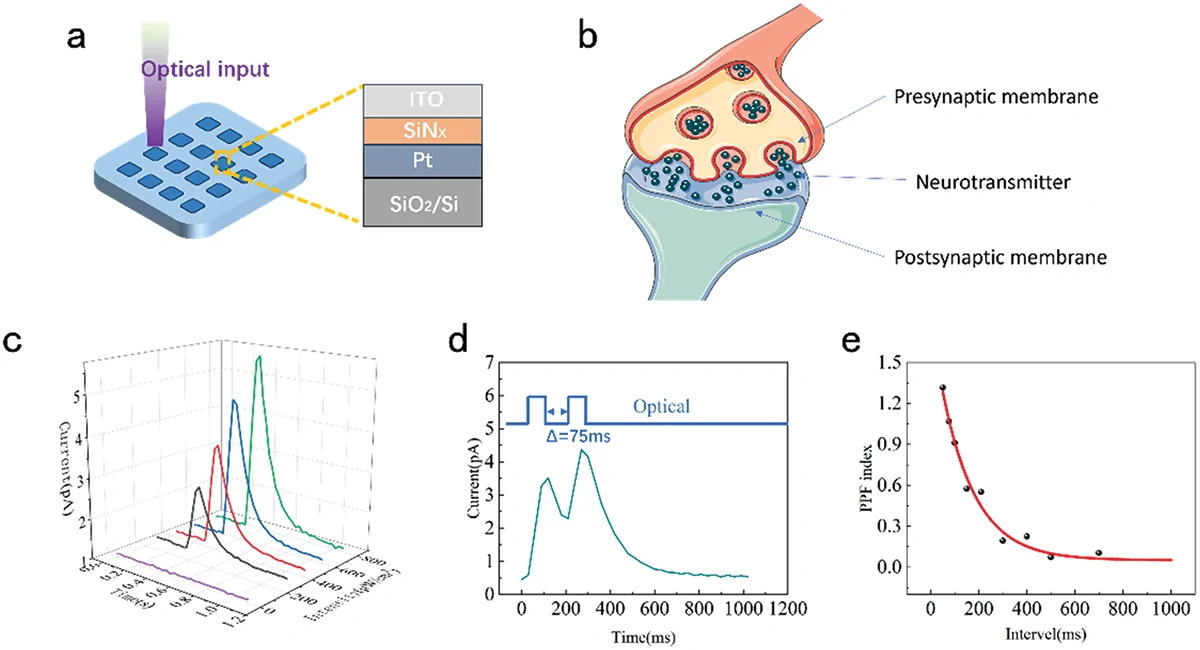
Short-term synaptic behaviors of ITO/SiN_x_/Pt device: (a) Device’s structure of ITO/SiN_x_/Pt. (b) Schematic diagram of EPSC generation for biological synapses. (c) EPSC characteristics of the ITO/SiN_x_/Pt device under UV stimulation with different light intensities (0, 290, 435, 580, 770 μW/cm^2^). (d) PPF behavior of the ITO/SiN_x_/Pt optoelectronic synapse. (e) Fitting curve of the PPF index versus pulse interval.

We investigated the short-term synaptic behaviors of ITO/SiN_x_/Pt optoelectronic synapses under UV illumination at a wavelength of 365 nm. During testing, a read voltage of 0.4 V was applied to the ITO electrode, while the Pt electrode was grounded. Figure 2(a) displays the two-terminal structure of ITO/SiN_x_/Pt. As shown in Figure 2(b), for biological synapses, EPSC is a representative short-term synaptic behavior, where action potentials trigger the release of neurotransmitters at the presynaptic terminal, resulting in a transient postsynaptic current [34,35,38]. The EPSC behavior of the ITO/SiN_x_/Pt optoelectronic synapses is demonstrated in Figure 2. Upon applying a 90 ms UV light pulse, the current increased and then decayed nonlinearly once the stimulation was removed. The free carriers between ITO and Pt electrodes can be regarded as analogs of neurotransmitters, and optical stimulation modulates their concentration, thereby altering the device’s conduction and inducing EPSC behavior. Under different light intensities of 0, 290, 435, 580, and 770 μW/cm^2^, the device exhibited a gradient EPSC response of 1.0, 2.7, 3.6, 4.6, and 5.6 pA, respectively, confirming its capability to effectively encode incident light intensity.

The illustrations in (b) were adapted from Servier Medical Art, licensed under CC BY 4.0.

PPF describes the enhanced postsynaptic response elicited by two consecutive synaptic stimuli [39]. The response current (ΔI) is defined as the difference between the peak photocurrent under illumination and the baseline current in the dark. As shown in Figure 2, when two identical UV pulses with an interval of 75 ms were applied to the device, the second stimulation induced a higher response (ΔI_2_) than the first (ΔI_1_), which is characteristic of typical PPF behavior. Figure 2(e) presents the relationship between the PPF index and the pulse interval (Δt) for the ITO/SiN_x_/Pt. The Δt was varied from 50 ms to 700 ms, and each measurement was repeated four times to obtain the average value. The results show that the PPF index (ΔI_2_ – ΔI_1_) decreases from 1.32 to 0.10 as Δt increases, indicating a time-dependent weakening of facilitation. This behavior fits well to a double-exponential decay function [40]:

$$\mathrm{PPF}\;\mathrm{index}=C_{0}+C_{1}e^{-\Delta t/\tau_{1}}+C_{2}e^{-\Delta t/\tau_{2}}$$

where $C_{0}$ is a constant term, $C_{1}$ and $C_{2}$ represent the initial facilitation amplitudes, $\tau_{1}$ and $\tau_{2}$ are the characteristic relaxation times for the fast and slow decay components, respectively. These results indicate a nonlinear relationship between the photocurrent and the interval of optical stimulation, demonstrating the ability of the ITO/SiN_x_/Pt synaptic device to encode the temporal features of optical signals.

**Figure 3.**
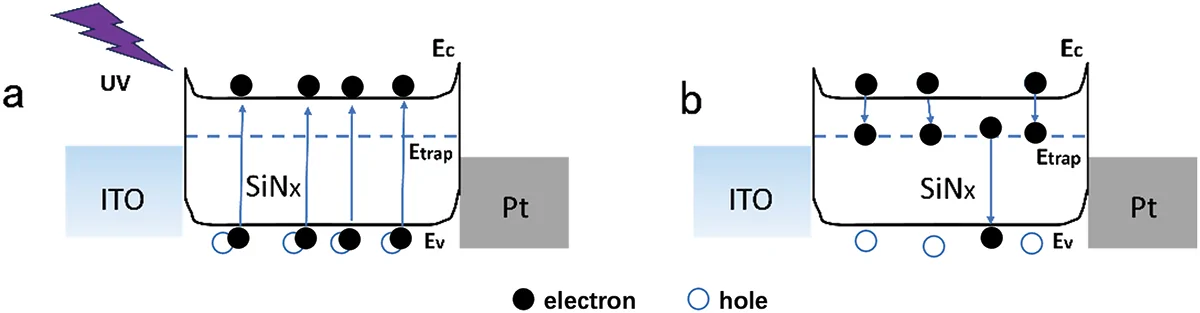
Working mechanism of the ITO/SiN_x_/Pt optoelectronic synapses: (a) Schematic band diagram of the ITO/SiN_x_/Pt device under UV illumination, showing the generation and separation of photo-induced electron–hole pairs. (b) Schematic band diagram of the ITO/SiN_x_/Pt device after the removal of UV light, showing the trapping of free electrons by the silicon dangling bonds.

The working mechanism of the ITO/SiN_x_/Pt synapses is illustrated in Figure 3. It is well known that SiN_x_ contains abundant silicon dangling bonds, which tend to trap the free carriers created by ultraviolet light [37,41,42]. Under UV illumination, electron–hole pairs are generated in the SiN_x_ film. Electrons are excited from the valence band to the conduction band, becoming free carriers and thereby increasing the device’s conductance. After the light is removed, the trap levels associated with the silicon dangling bonds capture some of the free electrons, inhibiting the recombination of electron–hole pairs. As a result, the current does not drop abruptly but decays nonlinearly over time. This mechanism enables the ITO/SiN_x_/Pt device to exhibit EPSC and PPF synaptic behaviors. The nonlinear decaying memory behavior is key for processing temporal signals in RC [11]. Therefore, we confirm the suitability of the ITO/SiN_x_/Pt optoelectronic synapses as virtual nodes in Si-RC system, functioning as sensing and preprocessing units of visual signals.

### Long-term synaptic behaviors of the SiN_x_ memristors

**Figure 4.**
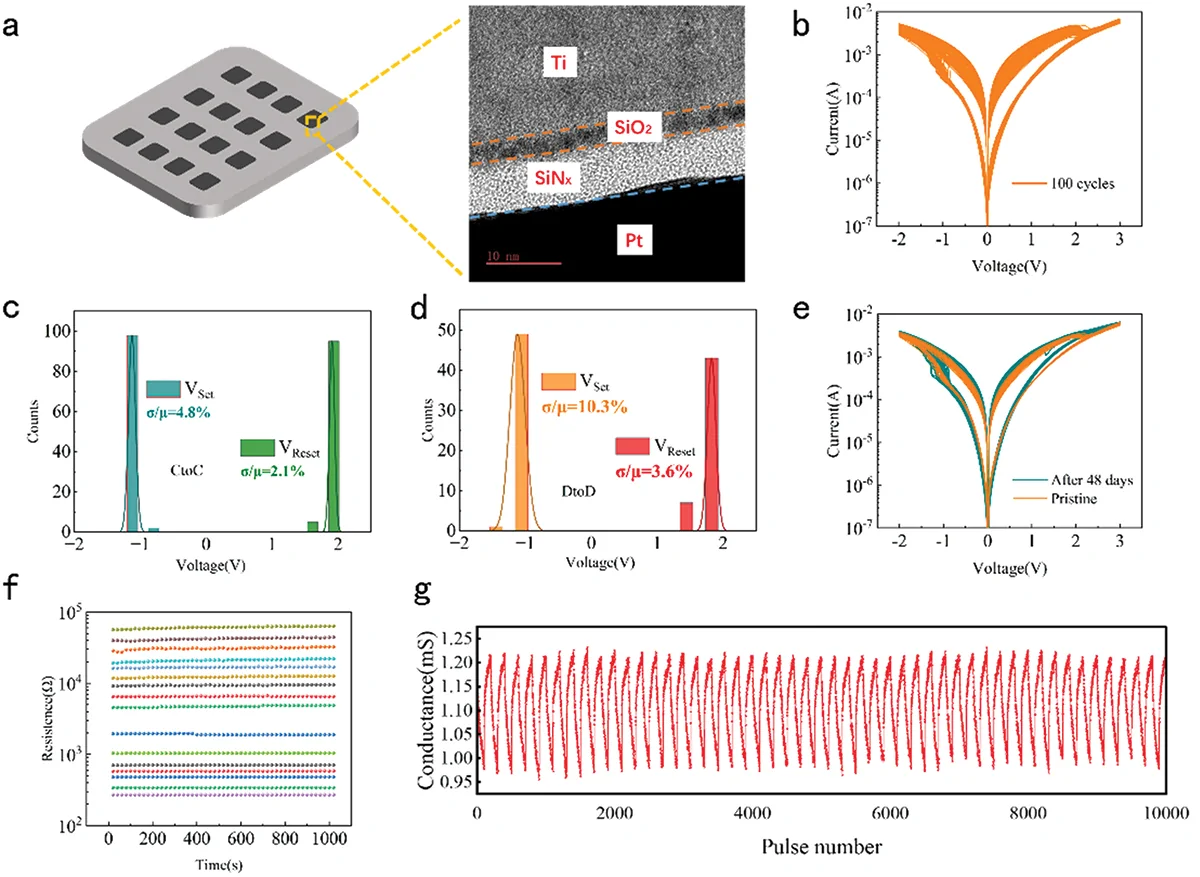
Electrical characteristics of the Ti/SiO_2_/SiN_x_/Pt memristor: (a) Device structure and TEM cross-sectional image of the Ti/SiO_2_/SiN_x_/Pt device, with a scale bar of 10 nm. (b) I-V curves for the device over 100 cycles. (c) The coefficient of variation for Set and Reset voltages over 100 cycles. (d) The coefficient of variation for Set and Reset voltages of 10 randomly selected devices. (e) I–V curves of the pristine device and after 48 days of testing. (f) Retention performance of 16 resistance states for 10^3^ seconds at 85 °C. (g) LTD/LTP characteristics of the Ti/SiO_2_/SiN_x_/Pt device after 10^4^ pulses, with potentiation and depression voltages setting to 1.15 V and −1.15 V.

Memristors can serve as trainable weights in the readout layer of Si-RC systems for training and inference. In this situation, memristors are required to exhibit non-volatility and continuously tunable conductance states [4,5]. Here, we proposed Ti/SiO_2_/SiN_x_/Pt memristor that exhibits uniform switching. In electrical measurements, the Pt electrode was biased while the Ti electrode was grounded. Figure 4(a) shows the schematic structure and cross-sectional transmission electron microscopy (TEM) image of the Ti/SiO_2_/SiN_x_/Pt device. A clear stacked structure is observed between the amorphous SiO_2_ and SiN_x_ layers, with a scale bar of 10 nm. As shown in Figure 4(b), the device demonstrates gradual resistive switching over 100 consecutive cycles without a forming process. The coefficient of variation (Cv) was employed to quantify the uniformity of the Ti/SiO_2_/SiN_x_/Pt memristors, which is defined as Cv = σ/μ, where σ is the standard deviation and μ is the mean. In the cycle-to-cycle (CtoC) tests, the Cv of V_Set_ and V_Reset_ over 100 switching cycles was as low as 4.8% and 2.1%, respectively (Figure 4). For device-to-device (DtoD) variation, we analyzed 10 randomly selected devices, and each was tested over 5 cycles, the Cv for V_Set_ and V_Reset_ was 10.3% and 3.6%, respectively (Figure 4). The results indicate high uniformity of Ti/SiO_2_/SiN_x_/Pt memristors, which is essential for large-scale memristor-based neuromorphic chips [43]. As shown in Figure 4(e), the device was retested after 48 days of initial test, and the results with 30 I–V sweeps closely overlapped with the initial curves, confirming the long-term stability of the Ti/SiO_2_/SiN_x_/Pt memristor. To evaluate its multilevel storage capability, incremental voltage pulses ranging from −2.2 V to 2.3 V were applied, with resistance readout taken after each voltage stimulation. As illustrated in Figure 4(f), the retention performance of 16 distinct resistance states for 10^3^ seconds at 85°C under 0.1 V read voltage, demonstrates the device’s multilevel characteristic.

The realization of long-term potentiation/depression (LTP/LTD) synaptic characteristics in memristors relies on their gradual conductance modulation [43,44]. As shown in Figure 4(g), the conductance states of the Ti/SiO_2_/SiN_x_/Pt device can be continuously modulated by up to 10^4^ consecutive voltage pulses, with each potentiation (−1.15 V, 500 ns) and depression (1.15 V, 500 ns) cycle consisting of 100 pulses. Quantitative analysis further reveals that the coefficient of variation Cv (σ/μ) of conductance during 1–10^4^ pulses is as low as 0.90%. Mechanism analysis (see Supplementary Figure S1) attributes the uniformity of LTP/LTD to the highly consistent formation and rupture of conductive paths related to Si dangling bonds. The material composition of the Ti/SiO_2_/SiN_x_/Pt memristors was analyzed by X-ray photoelectron spectroscopy (XPS), as shown in Supplementary Figure S2. The conduction mechanism of the Ti/SiO_2_/SiN_x_/Pt device was further investigated via positive voltage sweeps. As illustrated in Supplementary Figure S3, Schottky emission dominates the current transport in the low-resistance state (LRS), while the high-resistance state (HRS) conforms well to a combination of Schottky emission (at low voltages) and Poole–Frenkel emission (at high voltages). These results demonstrate strong potential of Ti/SiO_2_/SiN_x_/Pt memristors as trainable weights in Si-RC system.

### Movement recognition by Si-RC system

Based on the optoelectronic characteristics of the ITO/SiN_x_/Pt synapses and the LTP/LTD properties of the Ti/SiO_2_/SiN_x_/Pt memristors, we simulated a homogeneous Si-RC visual system by MATLAB for recognition of dynamic images, where the ITO/SiN_x_/Pt array functions as the reservoir layer and the Ti/SiO_2_/SiN_x_/Pt memristor array as the readout layer. The exhaust plume of a rocket contains abundant UV radiation, from which motion information can be extracted [2,45]. Based on this principle, the Si-RC visual system can sense the UV signals from the rocket plume and recognizes its movement directions. The rocket’s movement directions are classified into six types (up, down, left, right, approaching, away), as shown in Supplementary Figure S4. The proposed Si-RC system can directly sense and process optical images with multiple grayscale levels, eliminating the need for conventional image cropping and binarization procedures [4,46].

**Figure 5.**
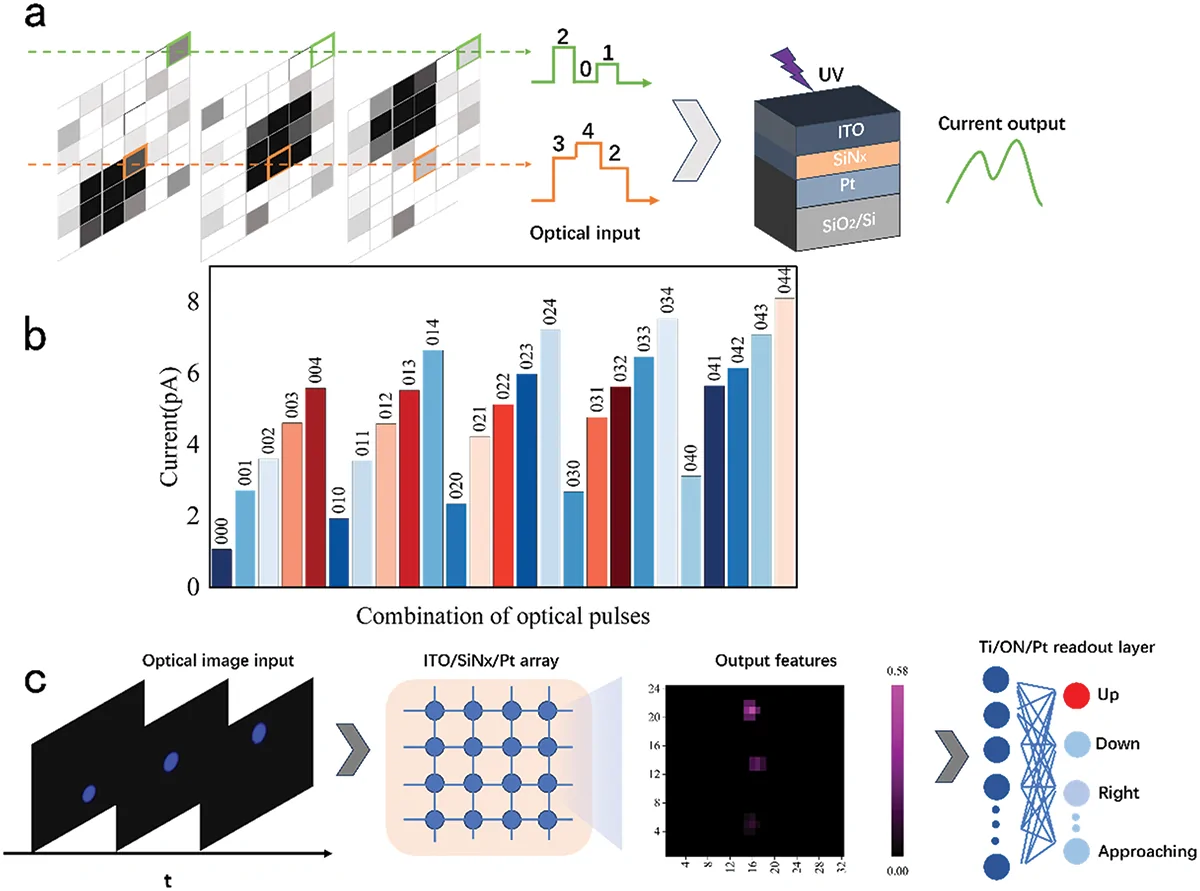
Recognition process for dynamic images by the Si-RC system: (a) Sensing and preprocessing of temporal signals by the ITO/SiN_x_/Pt synapses. (b) Photocurrents of the ITO/SiN_x_/Pt synapses to optical pulse combinations (from ‘000’ to ‘044’). (c) Schematic diagram of the working process of the Si-RC system.

The working process of the ITO/SiN_x_/Pt reservoir layer is illustrated in Figure 5(a). The dynamic image input consists of three image frames, each lasting 90 ms. The pixel values in the images are represented in five grayscale levels (‘0’ to ‘4’), corresponding to light intensities of 0, 290, 435, 580, and 770 μW/cm^2^, respectively. The dynamic images generate numerous temporal signals. For example, the signal ‘342’ represents three sequential UV pulses in an input sequence, with light intensities of 580, 770, and 435 μW/cm^2^, respectively. The photocurrent measured at the end of the entire input is used as the feature value, with each value corresponding to a temporal input. Therefore, the ITO/SiN_x_/Pt synapses encode the temporal features of the signals into current outputs. Figure 5(b) displays the photocurrents of selected optical pulse combinations (from ‘000’ to ‘044’), with additional results provided in Supplementary Figure S5. These results confirm that the input signals correspond to distinguishable currents which can be used as the temporal features. A 24 × 32 ITO/SiN_x_/Pt array is simulated to enable real-time sensing and preprocessing of dynamic images. As shown in Figure 5, the UV signals from the rocket illuminate the array, then each device in the array generates a photocurrent. As a result, the array produces a total of 24 × 32 currents, which serve as the overall features for the reservoir. In Si-RC system, the reservoir layer leverages intrinsic properties of ITO/SiN_x_/Pt optoelectronic synapses to process the dynamic images without training. Subsequently, the features from the ITO/SiN_x_/Pt array are passed to the readout layer based on Ti/SiO_2_/SiN_x_/Pt memristors for training and inference. The readout layer employs a three-layer MLP network with dimensions of 768 × 40, 40 × 15, and 15 × 6, respectively, where the network weights mapped onto the Ti/SiO_2_/SiN_x_/Pt memristors.

**Figure 6.**
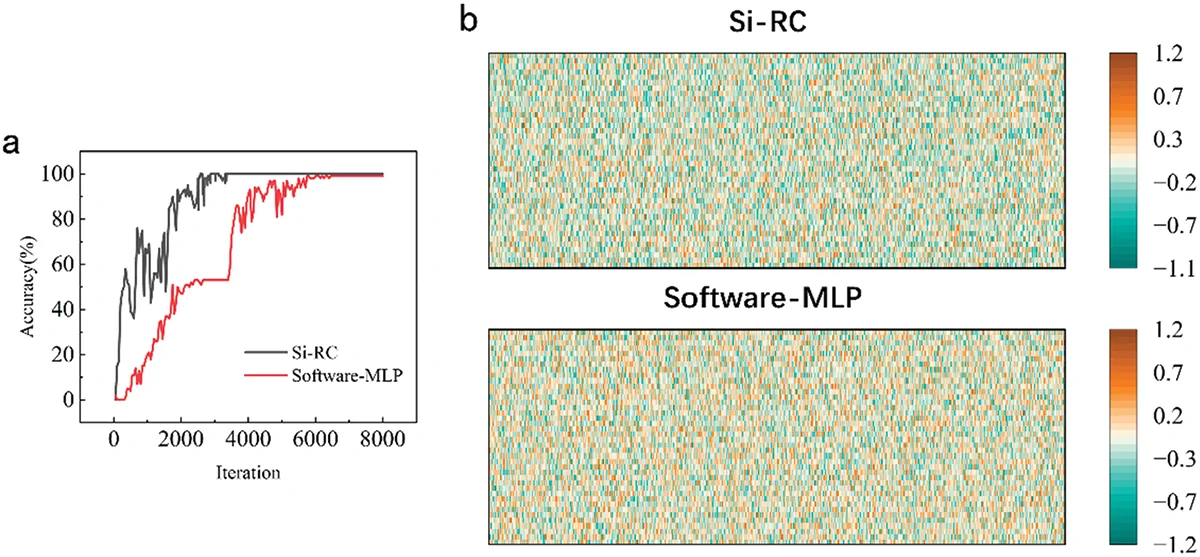
Comparison between Si-RC system and software-based MLP: (a) Comparison of recognition accuracy for rocket movement directions. (b) Weight color maps of Si-RC system and MLP.

As shown in Figure 6(a), after 3314 iterations, the Si-RC system achieves a stable recognition accuracy of 100% for rocket movement directions. However, the software-based MLP without image preprocessing achieved a recognition accuracy of 99% after 6450 iterations. The dataset consists of 900 training images and 100 testing images. The result demonstrates that the Si-RC system achieves a training speed 1.9 times faster than the MLP network, which can be attributed to the intrinsic preprocessing of optical dynamic images by ITO/SiN_x_/Pt reservoir layer. Figure 6(b) shows the weight color maps of the Si-RC system and the software-based MLP. In the Si-RC system, the weights are mapped onto the non-volatile Ti/SiO_2_/SiN_x_/Pt memristors. These weights can be stored in place after training and used for subsequent inference, minimizing data transfer processes.

## Conclusion

We developed silicon-based optoelectronic synapses and memristors. The ITO/SiN_x_/Pt optoelectronic synapses exhibit EPSC and PPF behaviors, while the Ti/SiO_2_/SiN_x_/Pt memristors demonstrate high uniformity, multilevel cell property, and reliable LTP/LTD characteristics. These two types of silicon-based devices form a homogeneous RC visual system for real-time sensing and recognition of dynamic images. The Si-RC system uses ITO/SiN_x_/Pt synapses as reservoir nodes to encode the temporal features of optical signals, and the readout layer composed of Ti/SiO_2_/SiN_x_/Pt memristors infer rocket movement directions. Compared to software-based MLP, the Si-RC system achieves 100% recognition accuracy and a 1.9-fold increase in training speed. The homogeneous silicon platform offers advantages including CMOS compatibility, reduced fabrication complexity, and improved scalability for large-scale integration. These advantages make it a promising platform for future neuromorphic vision systems and other integrated intelligent sensing applications.

### Device fabrication

The fabrication process of ITO/SiN_x_/Pt and Ti/SiO_2_/SiN_x_/Pt devices was as follows. Initially, 10 nm Ti was deposited on an SiO_2_/n-Si substrate via DC magnetron sputtering to serve as the electrode adhesion layer. Subsequently, the 100 nm Pt bottom electrode (BE) was deposited. The functional layer 7 nm SiN_x_ or 3 nm SiO_2_/6 nm-SiN_x_ for ITO/SiN_x_/Pt and Ti/SiO_2_/SiN_x_/Pt devices, respectively, was then deposited using plasma enhanced chemical vapor deposition (PECVD, Oxford Instruments, UK) at 300°C. The gas sources for SiN_x_ were SiH_4_, NH_3_ and N_2_; While those for SiO_2_, they were SiH_4_, N_2_ and N_2_O. Then, 100 nm ITO or Ti top electrode (TE) layer was deposited using DC magnetron sputtering. The TEs were patterned into square shapes with different areas using UV lithography and lift-off process. Finally, the fabricated Ti/SiO_2_/SiN_x_/Pt memristors were annealed at 350°C for 25 minutes in N_2_ atmosphere.

### Film characterization and device testing

XPS analysis was performed using an AXIS SUPRA+ system (Shimadzu Corporation, Japan). TEM characterization was performed using Quanta 3D FEG and Titan equipment (Thermo Fisher Scientific, USA). The electrical characteristics of the ITO/SiN_x_/Pt and Ti/SiO_2_/SiN_x_/Pt devices were measured using a B1500 semiconductor parameter analyzer (Agilent Technologies, USA). The optical inputs were controlled using a DC2200 LED driver (Thorlabs, USA).

## Supplementary Material

Supplemental Material

## Supporting information

The resistance switching mechanism of Ti/SiO_2_/SiN_x_/Pt memristors. XPS spectra of the Ti/SiO_2_/SiN_x_/Pt memristors. Conduction mechanism of Ti/SiO_2_/SiN_x_/Pt memristors. Simulated movement of the rocket in six directions. Currents of the ITO/SiN_x_/Pt synapses for light pulse combinations. (PDF)
