# Supplementary material for: Homogeneous reservoir computing system based on CMOS-compatible optoelectronic synapses and memristors
Source: Sci Technol Adv Mater. 2026 Aug 27;27(1):2718043. doi: 10.1080/14686996.2026.2718043 (PMC13577286; doi:10.1080/14686996.2026.2718043)
Supplement: Supplementary file 1 [file TSTA_A_2718043_SM5342.docx]

**Supporting Information**

Homogeneous reservoir computing system based on CMOS-compatible optoelectronic synapses and memristors

*Shaopeng Feng*^†^*, Kai Li*^†^*, Senhao Yan, Yunhao Luo, Xiaomin Cheng***, Xiangshui Miao*

School of Integrated Circuits, Hubei Key Laboratory for Advanced Memories, Wuhan National Laboratory for Optoelectronics, Huazhong University of Science and Technology, Wuhan 430074, China.

**Contents:**

Figure S1. The resistance switching mechanism of Ti/ON/Pt memristors.

Figure S2. XPS spectra of the Ti/ON/Pt memristors.

Figure S3. Conduction mechanism of Ti/ON/Pt memristors.

Figure S4. Simulated movement of the rocket in six directions.

Figure S5. Currents of the ITO/SiN_x_/Pt synapse for light pulse combinations.

The resistance switching mechanism of SiN_x_ memristors is widely believed to the conductive channel formed by Si dangling bonds (Si-DBs)^1–3^. The schematic of the resistance switching mechanism for Ti/SiO_2_/SiN_x_/Pt memristors is shown in Fig. S1. Differ from the ITO/SiN_x_/Pt optoelectronic synapses, the Ti/SiO_2_/SiN_x_/Pt memristors undergo an annealing process, forming SiO_y_N_z_ layer between SiO_2_ and SiN_x_, which serves as a reservoir for subsequent N ion migration and separation, and induced more Si-DBs within the SiN_x_ layer. When a forward electric field is applied between the Ti electrode (0 V) and the Pt electrode (-2 V), the negatively charged N ions separate with Si ions and gradually migrate toward the Ti electrode and are subsequently accommodated by the SiO_y_N_z_ layer. As Si-DBs accumulate in the resistive switching layer, a conductive channel gradually forms, transitioning the device from the high-resistance state (HRS) to the low-resistance state (LRS). Conversely, when a negative electric field is applied (Ti electrode at 0 V, Pt electrode at 3 V), N ions migrate and recombine with Si-DBs, causing the conductive channel to gradually break, and the device switches from LRS to HRS.

**Figure S1.** The resistance switching mechanism of Ti/SiO_2_/SiN_x_/Pt memristors.

The XPS of the annealed Ti/SiO_2_/SiN_x_/Pt memristors is shown in Fig. S2. In Fig. S2a, the Si 2p spectrum exhibits three peaks. The peak at 99.9 eV corresponds to the Si-Si bond which is attributed to the interconnection of partial Si-DBs^4^, the main peak at 101.3 eV represents the Si-N bond and the peak at 102.4 eV corresponds to the Si-O bonding^4,5^. In Fig. S2b, the N 1s spectrum shows a peak at 397.7 eV, corresponding to the Si-N bonding peak^2,6^. In Fig. S2c, the peak at 532.1 eV corresponds to the Si-O bond for O 1s spectrum^6^.

**Figure S2.** XPS spectra of the annealed SiN_x_ film: (a) Si 2p peak fitting. (b) N 1s peak fitting. (c) O 1s peak fitting.

To verify the conductive paths formed by Si-DBs, we measured the temperature dependence of LRS. Fig. S3a shows that the LRS resistance of the device decreases as the temperature increases from 298 K to 373 K, which corresponds to semiconductor behavior. The result rules out the possibility of metallic conductive filaments generated by the electrode within the SiN_x_, as the LRS resistance would increase with temperature if metallic filaments were formed^7,8^. The conduction mechanism of the Ti/ON/Pt device was further analyzed by positive voltage scanning. For the LRS, the fitting curve based on the Schottky emission model is shown in Fig. S3b. When an external electric field overcomes the metal/oxide interface potential barrier and injects electrons into the conduction band of the SiN_x_ switching layer, this process can be described by the following exponential relationship^9,10^:

$$J_{SE}\propto T^{2}exp\left( \frac{A\sqrt{E}}{T}-B \right)$$

where J represents the current density, T is the absolute temperature, and E is the electric field across the SiN_x_ layer.

For the HRS, the experimental data analysis indicates that the conduction mechanism in the low-bias region is consistent with Schottky emission (Fig. S3c). As the external electric field is increased to the high-bias region, the carrier conduction shifts to be dominated by Poole-Frenkel emission (Fig. S3d). This process corresponds to the thermal excitation of electrons from defect states (Si-DBs) within the SiN_x_ dielectric into the conduction band, and the current-field relationship can be expressed as:

$$J_{PF}\propto E\cdot exp\left( \frac{A\sqrt{E}}{T}-B \right)$$

where J represents the current density, T is the absolute temperature, and E is the electric field across the SiN_x_ layer.

**Figure S3.** Conduction mechanism of Ti/ON/Pt memristors: (a) Temperature dependence of the LRS resistance. (b) Carrier transport in the LRS fitting according to the Schottky emission model. (c) Schottky emission model fitting for the low-bias region of the HRS. (d) Poole-Frenkel emission model fitting for the high-bias region of the HRS.

Fig. S4a-f demonstrates six distinct movement directions of the rocket, which are: downward, upward, leftward, rightward, away, and toward. The movement process is set as three consecutive UV images.

**Figure S4.** Simulated movement of the rocket in six directions: (a) Downward. (b) Upward. (c) Leftward. (d) Rightward. (e) Away. (f) Toward.

Fig. S5a-d show the response current values of the ITO/SiN_x_/Pt device to light pulses ranging from ‘100’ to ‘444’. Each light pulse combination corresponds to a specific current, thus allowing the optoelectronic synapse to encode the temporal information of the light into characteristic current values. These characteristics will be used for training the readout layer.

**Figure S5.** Currents of the ITO/SiN_x_/Pt synapse for light pulse combinations: (a) from ‘100’ to ‘144’. (b) from ‘200’ to ‘244’. (c) from ‘300’ to ‘344’. (d) from ‘400’ to ‘444’.

**Reference**

(1)	Bai, Y.; Duan, Y.; Qian, M.; Zhu, S.; Ma, X.; Yang, Y.; Gao, H. Temperature Dependence of Electrical Properties and Conduction Mechanism of SiN-Based Resistive Random Access Memory. *physica status solidi (a)* **2023**, *220* (21), 2300398. https://doi.org/10.1002/pssa.202300398.

(2)	Kwon, D. E.; Kim, Y.; Kim, H. J.; Kwon, Y. J.; Woo, K. S.; Yoon, J. H.; Hwang, C. S. Bipolar Resistive Switching Property of Si3N4−x Thin Films Depending on N Deficiency. *J. Mater. Chem. C* **2020**, *8* (5), 1755–1761. https://doi.org/10.1039/C9TC05336K.

(3)	Yang, M.; Wang, H.; Ma, X.; Gao, H.; Wang, B. Effect of Nitrogen-Accommodation Ability of Electrodes in SiNx-Based Resistive Switching Devices. *Applied Physics Letters* **2017**, *111* (23), 233510. https://doi.org/10.1063/1.4996618.

(4)	Pavlyák, F.; Bertóti, I.; Mohai, M.; Biczó, I.; Giber, J. AES and XPS Characterization of SiNx Layers. *Surface and Interface Analysis* **1993**, *20* (3), 221–227. https://doi.org/10.1002/sia.740200306.

(5)	Lu, P.; Mu, W.; Xu, J.; Zhang, X.; Zhang, W.; Li, W.; Xu, L.; Chen, K. Phosphorus Doping in Si Nanocrystals/SiO2 Multilayers and Light Emission with Wavelength Compatible for Optical Telecommunication. *Sci Rep* **2016**, *6* (1), 22888. https://doi.org/10.1038/srep22888.

(6)	Kim, S.; Kim, H.; Hwang, S.; Kim, M.-H.; Chang, Y.-F.; Park, B.-G. Analog Synaptic Behavior of a Silicon Nitride Memristor. *ACS Appl. Mater. Interfaces* **2017**, *9* (46), 40420–40427. https://doi.org/10.1021/acsami.7b11191.

(7)	Privitera, S.; Bersuker, G.; Butcher, B.; Kalantarian, A.; Lombardo, S.; Bongiorno, C.; Geer, R.; Gilmer, D. C.; Kirsch, P. D. Microscopy Study of the Conductive Filament in HfO2 Resistive Switching Memory Devices. *Microelectronic Engineering* **2013**, *109*, 75–78. https://doi.org/10.1016/j.mee.2013.03.145.

(8)	Yang, Y. C.; Pan, F.; Liu, Q.; Liu, M.; Zeng, F. Fully Room-Temperature-Fabricated Nonvolatile Resistive Memory for Ultrafast and High-Density Memory Application. *Nano Lett.* **2009**, *9* (4), 1636–1643. https://doi.org/10.1021/nl900006g.

(9)	Lim, E. W.; Ismail, R. Conduction Mechanism of Valence Change Resistive Switching Memory: A Survey. *Electronics* **2015**, *4* (3), 586–613. https://doi.org/10.3390/electronics4030586.

(10)	Chiu, F.-C. A Review on Conduction Mechanisms in Dielectric Films. *Advances in Materials Science and Engineering* **2014**, *2014*, e578168. https://doi.org/10.1155/2014/578168.
